# Construction of a Fab Library Merging Chains from Semisynthetic and Immune Origin, Suitable for Developing New Tools for Gluten Immunodetection in Food

**DOI:** 10.3390/foods12010149

**Published:** 2022-12-28

**Authors:** Eduardo Garcia-Calvo, Aina García-García, Santiago Rodríguez, Sergio Farrais, Rosario Martín, Teresa García

**Affiliations:** 1 Departamento de Nutrición y Ciencia de los Alimentos, Facultad de Veterinaria, Universidad Complutense de Madrid, 28040 Madrid, Spain; 2 Servicio de Medicina Digestiva, Hospital Universitario Fundación Jiménez Díaz, 28040 Madrid, Spain

**Keywords:** gluten, prolamins, celiac disease, phage display, ELISA, immune library, semi-synthetic library

## Abstract

The observed increase in the prevalence of gluten-related disorders has prompted the development of novel immunological systems for gluten detection in foodstuff. The innovation on these methods relies on the generation of new antibodies, which might alternatively be obtained by molecular evolution methods such as phage display. This work presents a novel approach for the generation of a Fab library by merging semi-synthetic heavy chains built-up from a pre-existent recombinant antibody fragment (dAb8E) with an immune light chain set derived from celiac donors. From the initial phage population (10^7^ candidates) and after three rounds of selection and amplification, four different clones were isolated for further characterization. The phage Fab8E-4 presented the best features to be applied in an indirect ELISA for the detection of gluten in foods, resulting in improved specificity and sensitivity.

## 1. Introduction

Gluten is defined by Codex Alimentarius as ethanol-soluble but water and 0.5 M sodium chloride insoluble protein fractions, that are present in some cereal endosperms like wheat, rye, barley, spelt, and kamut [1]. Gluten proteins are divided into two fractions by their solubility in a 60% ethanol/water solution: soluble gliadins and insoluble glutelins. These chemical features result in different technological properties of dough, as gliadins are related to viscosity and extensibility, while glutelins contribute to strength and stability [2]. The extraordinary physical-chemical stability of gluten protein fractions (like gliadin) results in slow partial digestion and high permanence in the gut, that is related to its pathogenicity: undigested gliadin peptides increase gut permeability and have specific binding to CXCR3 chemokine receptor, with a subsequent release of zonulin (a modulator of tight junctions). This occurs in all individuals; however, it can lead to inflammatory processes in sensitized persons, like those presenting the human leukocyte antigen (HLA)-DQ2 and DQ8 haplotype [3].

There are several gluten-related diseases classified by etiopathology. Firstly, those classified as allergic diseases like baker’s asthma (respiratory allergy), food allergy, contact urticaria and, WDEIA (wheat-dependent exercise-induced anaphylaxis) [4]. The second group clusters autoimmune diseases like celiac disease, the best known, caused by a strong immune response against gluten-processing self-transaminases, ending in a severe destruction of gut tissue. There are other diseases included in this group, as gluten ataxia and dermatitis herpetiformis [5]. The third group is called non-celiac gluten sensitivity for those situations that do not fit in the previous groups. It seems that they could be the most prevalent diseases and their molecular etiology is not completely elucidated but, some studies show a core implication of the innate immune system [6].

Due to the health impact of gluten-related diseases, several countries have developed gluten labeling legislation, most of them following Codex Alimentarius limit of 20 parts per million (ppm) to be considered a gluten-free product [7].

Several analytical methods have been developed to detect and quantify gluten in foodstuffs, including PCR [8], chromatography and mass spectrometry [9]. However, the immunological techniques, including ELISA methods [10], are the most widely used. The development of novel and more reliable gluten immune-detection methodologies are coupled with antibody discovery. There are three main technologies: polyclonal antibodies from animal serum, monoclonal antibodies from hybridomas, and recombinant antibodies in the context of molecular evolution strategies [11].

The first methods were developed on the basis of polyclonal antibodies derived from rabbit IgGs [12] and chicken IgYs [13] and, nowadays they are still in use. The revolution of monoclonal antibodies also had a great impact on this field. Three hybridoma derived antibodies represent the main reactants for the most widely used gluten-detection methods: 401.21 [14], R5 [15] and G-12 [16]. A sandwich ELISA based on R5-mAb is the golden standard for gluten detection in food validated by AACI (American Association of Cereal Chemists International), AOAC International, and Codex Alimentarius [17].

Although polyclonal and monoclonal antibodies are widely applied for gluten detection in food, the development of new recombinant antibodies could bring new insights to the field. Recombinant antibodies can be developed in shorter, cheaper, and simpler processes than monoclonal “classical” antibodies. Unlike mono and polyclonal antibodies, recombinant antibody technology allows complete animal-free development and production of specific binders for immunoassays [18].

Antibodies are plasmocite secreted proteins composed of four polypeptide chains, classified by their molecular weight in two heavy and two light chains. Light chains are composed by a constant and a variable domain and, in humans, they are coded by genes that reside in two different chromosomes (resulting in two kinds of light chains, kappa and lambda). Heavy chains are composed of a variable domain and three or four constant domains (the case of human IgE or avian antibodies). The variable domains have two topological domains; three juxtaposed loops called complementary determining regions or CDRs, that drive the main antigen-antibody interaction and, in between, rigid and conserved β-barrels, the frameworks regions (FRs) [19]. Camelids show a unique antibody structure, with two neighbouring single heavy chains [20]. Thanks to the development of recombinant expression of antibodies, it is possible to produce non-natural engineered antibody fragments and natural constructions, like Fabs (conformed by the light chain attached to the variable and first constant domain of heavy chain), dAbs (the variable domain of heavy chain) and many combinations of the elements mentioned above.

The final years of the 20th century meant a new revolution on antibody development thanks to the works of Winter and Smith [21], that introduced the concept of directed molecular evolution. This method goes beyond the recombinant expression of antibodies. It encloses three main steps: genesis of diversity, selection and amplification of high-affinity antibodies against a target antigen. One to ten binders can be chosen from initial 10^7^ antibodies, after 3 to 6 rounds of selection. After selection, these binders become dominant within the population, and their genotype is coupled with their phenotype (thanks to phage, ribosome or cell surface display of molecules). There are several examples of the application of directed evolution for the elucidation of molecular mechanisms of celiac and related diseases, using phage display. They include immune libraries, but also naïve [22] and peptide libraries [23]. Although the clinical research has been the main application, there have been several projects aimed to develop antibodies for gluten detection in food using camelid antibodies. Immunized llamas were used for the construction of a phage-displayed VHH library, with the objective of obtaining a capture antibody for gluten detection by sandwich-ELISA [24]. Moreover, a naïve llama library was used for the development of a novel gluten detection methodology (cDNA display mediated immuno-PCR) [25].

The present work is the continuation of a novel strategy for isolation of high affinity antibodies against gluten [26], where a semi-synthetic library based on human VH3 germline, was confronted to a peptide proposed to be a consensus HLA-presented peptide of digested gluten. From this process, a suitable candidate was selected (dAb-8E) and used as the specific reagent for the development of a phage-ELISA method for gluten detection in foodstuff.

Although the antibody specificity is mainly the result of the interaction between the V-region with the antigen, constant domains also affect to antigen binding, despite they are not participating on the physical antibody-antigen interaction [27]. The constant domains influence in segmental flexibility and inter-antibody associations, fixing the conformational signaling through the V_H_-C_H1_ domains, so that, structural and kinetic constraints are imposed to the contact surface [28]. So, it was hypothesized that the transformation of the dAb-8E into a Fab could enhance its properties. Therefore, the main objective of this work was the generation of a new antibody library merging chains of two different origins: a light-chain library derived from plasmocites isolated from celiac patients, and heavy chains derived from the dAb8E coding sequence transformed into human heavy-chains (IgG1, IgG2, IgG3 and IgG4).

## 2. Materials and Methods

### 2.1. Bacterial Strains and Growth Media

*Escherichia coli* XL1-Blue strain (recA1 endA1 gyrA96 thi-1 hsdR17 supE44 relA1 lac [F proAB lacIqZΔM15 Tn10 (Tetr)]) (Agilent^©^, Santa Clara, CA, USA, #ref 200150) was used for cloning, building the libraries and producing phage-displayed Fabs. Electrocompetent cells were in-house produced by growing the mentioned strain in super-broth medium (SB: 30 g/L tryptone, 20 g/L yeast extract, 10 g/L MOPS pH 7) and concentrated by sequential cycles of centrifugation (2218× *g*, 4 °C) and resuspension in 10% glycerol (mL/mL) (PanReac AppliChem^©^, Monza, Lombardy, Italy, CAS: 56-81-5) diluted in water. After electroporation, cells were grown in SOC medium (Invitrogen™-Thermo Fisher^©^, Waltham, MA, USA, #ref 15544-034). For DNA extraction before sequencing, bacteria were grown in Luria Broth (LB: 10 g/L tryptone, 5 g/L yeast extract, 10 g/L NaCl). Agar plates were prepared with a 15 g/L agar concentration.

### 2.2. DNA Isolation, Quantification, and Restriction

All DNA products used for library construction were isolated by gel electrophoresis using UltraPure™ Low Melting Point agarose (Thermo-fisher^©^, #ref 16520050), and purified with NucleoSpin^®^ gel DNA clean-up columns (Machery-Nagel^©^, Allentown, PA, USA, #ref 740609). DNA was quantified in a Qubit^®^ Fluorometer (Invitrogen Thermo-fisher^©^), and its quality was measured with a NanoDrop ND-1000 spectrophotometer (NanoDrop Technologies Inc., Montchanin, DE, USA). Restriction enzymes *Sac*I #ref R0156, *Xba*I #ref R0145, *Xho*I #ref R0146 and *Spe*I #ref R0133 and T4 DNA ligase #ref M0202, were purchased from New England Biolabs^©^ (Ipswich, MA, USA).

### 2.3. Semisynthetic Heavy Chain Sub-Library Construction

The original sequence of dAb-8E [26] had an intra-sequence *Xho*I restriction site that required removal for proper cloning by a single-base mutation with Agilent^©^ QuickChange II kit (Santa Clara, CA, USA, ref #200523). Once the single-point mutation was verified by sequencing, the heavy chains for library construction were built by a two-step PCR process. Forward primer VH135 and reverse primers ovlp-conga and ovlp-cong4 (Table S1) were used in the first round of PCR to obtain the VH domain of the mutated dAb-8E. This first step incorporated a *Xho*I site at 5′ end, and, the initial nucleotides of CH1 domains at the 8E-FR4 end (IgG1, IgG2 and IgG3 sequences hybridized with ovlp-conga primer, and IgG4 sequence with ovlp-cong4 primer). During the second round of PCR, the first-round amplicons were fused to the remaining CH1 sequences by overlap extension PCR with the forward primer VH135ext and the reverse primers CG1Zext, CG2aext, CG3aext, and CG4aext (Table S1) [29], generating the coding sequences of the heavy chains to be incorporated into the final Fab-repertoire, and a *Spe*I site at 3′ end for clonation.

### 2.4. Immune Light Chain Sub-Library Construction

Two patients newly diagnosed with celiac disease were selected as donors for this work in the digestive medicine service of Fundación Jiménez Díaz Hospital (Madrid, Spain). The humoral response against gliadin was quantified using ELISA tests: α-Gliatest S Chromo IgA (Eurospital, Trieste, Italy, ref #910796) and α-Gliatest S Chromo IgG (Eurospital, ref #910896). After acceptance of the informed consent, 350 mL of peripheral blood was obtained from each donor and mononuclear cells were isolated using a Ficoll gradient (Merck, Darmstadt, Germany, CAS #26873-85-8). Total RNA was extracted by a standard method with TRIzol LS reagent (Invitrogen™-Thermo Fisher^©^, ref #10296010). Once the RNA quality and quantity was checked by Agilent^© 2022^ Bioanalyzer (ref #G2939BA), it was used as template in reverse transcription reactions (SuperScript IV^®^, Invitrogen™- Thermo Fisher^©^, ref #18091050). Double stranded DNA encoding the light chain repertoire was amplified using the collection of primers described by Barbas et al. [29] (Table S2). Finally, the purified amplicons were re-amplified in order to add the *Sac*I/*Xba*I restriction sites needed for cloning in the pComb3X vector.

### 2.5. Fab-Library Construction

Library construction was developed as described by Burton et al. [30] with minor modifications. Light chain amplicons (1.5 µg) and pComb3X vector were *Sac*I/*Xba*I double-digested and gel purified. Digested inserts and linearized vector were ligated (3:1 molar ratio) at 16 °C for 16 h with T4 DNA ligase. The resulting ligation was ethanol precipitated, re-suspended in 20 µL of water and transformed into high-efficiency electrocompetent *E. coli* XL1-Blue. Subsequently, 3 mL of SOC medium was added, incubated, and shaken at 250 rpm for 1 h at 37 °C. At this point, a small aliquot of the culture was removed and titrated in LB agar plates containing 100 µg/mL of carbenicillin. The rest of the culture was transferred to 12 mL of LB broth with 20 µg/mL of carbenicillin and 10 µg/mL of tetracycline and incubated for an additional hour. Afterwards, the culture was expanded to 100 mL of LB (carbenicillin at 50 µg/mL and tetracycline at 10 µg/mL) and grown overnight. To ensure the proper performance of the cloning process, the plasmid DNA from 30 individual clones was sequenced (Table S3).

Phagemid DNA containing the immune light chain repertoire was purified and *Xho*I/*Spe*I digested for heavy chain insertion. A total of 1.5 µg of the previously obtained light chain sub-library were *Xho*I/*Spe*I digested and the linearized phagemids gel purified. Subsequently, the semi-synthetic heavy chain sub-library was cloned following the steps described above for the light chain. The resulting ligation was ethanol precipitated and transformed into *E. coli* XL1-Blue. After electroporation, 5 mL of recovering SOC medium was added, and shaken at 250 rpm at 37 °C for 1 h. A small aliquot was removed, ten-fold serial diluted and plated into SB plates (containing 100 µg/mL of carbenicillin) in order to calculate the repertoire size of the library. Ten milliliters of SB (containing 20 µg/mL of carbenicillin and 10 µg/mL of tetracycline) was added to the remaining culture and incubated under the same conditions for 1 h. At this point, carbenicillin concentration was increased to 50 µg/mL and the culture was incubated for one additional hour. Then, 2 mL of VSCM13 helper phage (10^12^–10^13^ pfu/mL) was added, and the culture was transferred to 200 mL of SB (containing 50 µg/mL of carbenicillin and 10 µg/mL of tetracycline) and shaken at 300 rpm for 2 h. Kanamycin was added to a concentration of 70 µg/mL and the culture incubated overnight with shaking (300 rpm) at 37 °C.

The following day, the culture was centrifuged at 2218× *g* for 20 min at 4 °C. The phagemid DNA obtained from the pelleted cells constituted the DNA library, and the phage-Fab library was isolated from the culture supernatant by PEG-NaCl precipitation. The supernatant (200 mL) was mixed with 8 g of PEG-8000 (Sigma^©^, Burlington, MA, USA, #ref 89510) and 6 g of NaCl (Sigma^©^, #ref S3014), incubated on ice for 1 h and centrifuged at 10,016× *g* for 20 min at 4 °C. The supernatant was discarded and the phage-antibodies were resuspended in 2 mL of 1% BSA (NZYtech^©^, Lisbon, Portugal, #ref MB04602) in PBS (137 mM NaCl, 2.7 KCl, 10 mM Na_2_HPO_4_, 1.8 mM KH_2_PO_4_, pH 7.4).

### 2.6. Selection of Gliadin-Binding Phage-Fab Variants by Panning

Four wells from a 96-well immunoplate were coated for 1 h at 37 °C with 100 µL of gliadin-PWG at 10 µg/mL in 0.1 M carbonate/bicarbonate buffer pH 9.6. The coating solution was removed, and after three washing steps with PBS, 400 µL of blocking solution (3% BSA in PBS) was added to each well. After incubating the plate at 37 °C for 1 h and performing ten washes with PBS, 50 µL of the phage-antibody library was added per well and the plate was incubated at 37 °C for 2 h. Non-binding phages were washed-away with five washing steps with PBS-T (PBS containing 0.05% of Tween20). Gliadin binding phage-antibodies were eluted with 50 µL of 0.1 M glycine-HCl-1% BSA (pH 2.2) by scratching the bottom of the well with a cut pipette tip for 10 min. Finally, the recovered phages were neutralized with Tris-Base 2 M (Sigma^©^, #ref T0440) to pH 7.

The eluted phage-antibodies were used to infect a 2 mL culture of *E. coli* XL1-Blue grown in SB (10 µg/mL of tetracycline) at OD_600_ = 0.8–0.9. After 15 min of infection, a small aliquot was ten-fold diluted and plated in order to calculate the output tittering. Afterwards, 6 mL of SB (containing 20 µg/mL of carbenicillin and 10 µg/mL of tetracycline) was added to the infected culture and further incubated at 37 °C for 1 h (250 rpm). Carbenicillin concentration was increased to 50 µg/mL and the culture incubated for 1 h at 37 °C with shaking at 270 rpm. Then 1 mL VSCM13 helper phage was added, and the culture expanded to 100 mL of SB (containing 50 µg/mL of carbenicillin and 10 µg/mL of tetracycline) and shaken for 2 h at 37 °C (300 rpm). Kanamycin was added to a concentration of 70 µg/mL and the culture was incubated overnight at 37 °C (300 rpm). This amplified phage-antibody population obtained from the first round of panning was tittered and used as input for the second round of selection against gliadin. A total of three rounds of selection were performed following the protocol described above, but with an increase in the number of washes for the removal of non-specific phages: 5, 10 and 15 for the first, second, and third round of panning, respectively. In addition, four wells were used in the first round, then the number was reduced to two in following rounds.

### 2.7. Preparation of Antigens

Gliadin-PWG was used as reference material for gluten detection in foods (Arbeitsgemeinschaft Getreideforschung (AGF) e.V. DIGeFa GmbH, Detmold, Germany). Following the supplier’s recommendation, it was dissolved in a 60% ethanol/water solution at 100 µg/mL and stored at room temperature protected from light. Gluten-like proteins were also extracted from different cereal matrices such as wheat, rye, barley, corn, and rice, as well as a binary mixture of wheat, rye, and barley kernels in a rice-based gluten-free matrix. Ten food products were tested (Table S4) following the protocols for extraction, analysis and result confirmation as previously described in García-García et al. [24]. Gluten-like proteins were extracted from 250 mg of the finely ground sample in 10 mL of a 60% ethanol (or 200 mg in 1 mL of 50% isopropanol solution in the case of Western blot analysis), followed by shaking for 30 min at room temperature and centrifugation at 867× *g* for 15 min at room temperature. The ethanol extracts were transferred to a clean vial and stored at room temperature protected from light until analysis.

### 2.8. Small and Medium Scale Individual Clone Induction for Analysis

Individual colonies isolated from the tittering output plates were grown in 4 mL of SB medium (50 µg/mL of carbenicillin) and incubated at 37 °C until OD_600_ reached 0.9–1. At this moment, 40 µL of VSCM13 helper phage preparation (10^12^–10^13^ pfu/mL) was added and incubated a 37 °C for 2 h with shaking. After helper infection, 70 µg/mL of kanamycin was added and the phage-antibodies produced in the supernatant during an overnight incubation at 37 °C. Selection of gliadin binding phage-antibody candidates was performed by indirect ELISA of supernatants. The phage-antibodies from the selected clones were produced using the same protocol but on a larger scale (100 mL of SB-carbenicillin) and the phage-Fabs were PEG-NaCl purified as described in the panning protocol. The phage preparation was tittered using the classical top-agar methodology, presenting 4–6 × 10^12^ phage particles per milliliter.

### 2.9. Indirect Phage-Fab ELISA

Gluten proteins extracted from flour samples or gliadin-PWG were diluted in coating buffer (0.1 M carbonate/bicarbonate buffer, pH 9.6). One hundred microliters of the prepared extracts were added per well and the plate was sealed and incubated for 1h at 37 °C. The coating solution was shaken out and 200 µL blocking solution (3% BSA in PBS) was added per well and, the plate was incubated for 1 h at 37 °C. Following 10 washing steps with PBS, 100 µL of diluted phages were added and plates incubated 2 h at 37 °C. The non-binding phages were washed away 10 times with PBS. Then, 100 µL of the secondary antibody (anti-phage protein VIII HRP conjugated from Sinobiological^©^, Beijing, China, #ref 11973) diluted 1:5000 in blocking buffer was added. The plate was incubated for 1 h at 37 °C and PBS washed 10 times. One hundred microliters of TMB (Sigma^©^, #ref T0440) was added, and the reaction was stopped after 15–20 min with 50 µL of a diluted sulphuric acid solution. Absorbance readings were performed at 450 nm wavelength (FluoStar Optima™ from BMG labtech^©^, Ortenberg, Germany).

### 2.10. Western Blot Analysis of Gliadin and Gluten-Containing Cereals

The different alcohol-soluble fractions of the commercial gliadin and the gluten-containing cereals were separated in two identical acrylamide gels (AnyKD precast-gel from Bio-Rad^©^, Hercules, CA, USA, #ref 4569033) under denaturing conditions at 90 V using NZYcolour Protein Marker II (NZYtech^©^, #ref M090). After electrophoresis, one of the gels was stained with Coomassie blue and the protein bands of the twin gel were transferred to a PVDF membrane at 100 V for 1.5 h. The PVDF membrane was blocked overnight with 3% BSA in TBS (50 mM Tris HCl, 150 mM NaCl, pH 7.5) before incubation with a 1:45 dilution (TBS-BSA 1%) of the PEG-NaCl precipitated phage-Fab8E-4 for 2 h at 37 °C in a rocking platform. Membrane was rinsed 5 times with TBS and incubated for 1 h at 37 °C with the anti-M13proteinVIII-HRP secondary antibody diluted 1:5000 in blocking solution. The image was revealed after the addition of a chemo-luminescent reactive (Clarity™ ECL Western blotting substrate from Bio-Rad^©^, #ref 1705061) in a ChemiDoc Imaging System (Bio-Rad^©^).

### 2.11. Sequencing and Molecular Modeling of Fab8E-4

DNA from the isolated gliadin-binding phage-Fab clones was extracted with the plasmid mini-prep (GenElute™ plasmid miniprep kit from Sigma© #PLN70), and the phagemids sequenced by Sanger method (Eurofins genomics^©^, Luxemburg, Luxemburg) using the primers ompAseq and g-back (Table S3) for light and heavy chain reading, respectively [29].

The antibody coding sequences were searched using the IMGT^®^/V-QUEST alignment tool [31] and the IgBLAST tool from NCBI [32]. Furthermore, amino acid sequence from Fab8E-4 was used to obtain a predictive model of the Fab structures using the sAb Pred server [33]. Once the Fab structure was generated, paratope and epitope prediction were calculated using the above mentioned server for which antigen structures were modeled with Alpha-fold [34].

## 3. Results and Discussion

### 3.1. Semi-Synthetic Heavy Chain Sub-Library Construction

**Figure 1:**
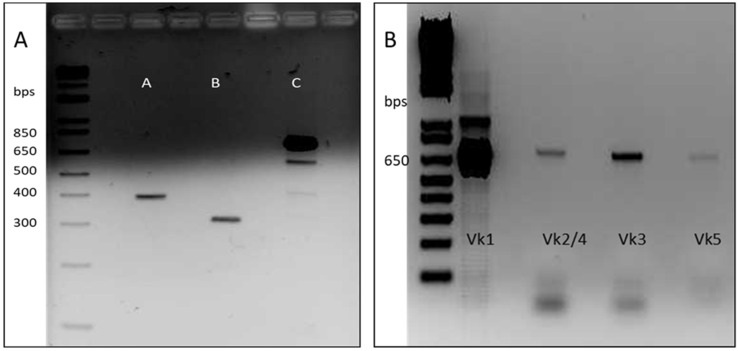
Agarose gel containing coding fragments for antibody chains to be cloned for library construction. (**A**) Different constructs that built the semi-synthetic heavy chains. Ladder: 1 kb plus Invitrogen™ (#ref 10787018); lane A: dAb-8E amplified sequence from the first round of PCR (VH-392 bp); lane B: CH1 amplified sequence from the first round of PCR (CH-300 bp); lane C: construct with the heavy chains obtained from the second round of PCR (SOE-PCR) prepared for Fab library cloning (H-680 bp). (**B**) Electrophoretic analysis of the human kappa chain genes amplified by PCR (Ladder: 1 kb plus Invitrogen©).

The main objective of this work was the development of a Fab library constructed by a two-step cloning process consisting of cloning firstly the immune-light chains and subsequently the semi-synthetic heavy chains. For Fab heavy chain construction, the sequence of a single domain antibody (named dAb-8E) previously obtained by our group against gluten was used [25]. Before cloning, the dAb-8E sequence was mutated in order to eliminate an intra-sequence restriction site that could originate the formation of wrong constructs. Since dAb sequence lacks constant heavy chain region, the Fab sequences were built-up from the dAb-8E sequence conforming the variable region, and the CH1 constant region was added by SOE-PCR. A first round of PCR was performed to obtain the dAb and CH1 sequences originating the intermediate constructs of 392 and 300 bp, respectively (Figure 1A-lanes A and B). The gel purified DNA fragments were used as templates for the second round of PCR that allowed the overlap of the dAb-8E with the CH1 coding sequences, obtaining the heavy-chain coding constructs (Figure 1A-lane C) of approximately 680 bp, prepared for next step.

### 3.2. Immune Light Chain Sub-Library Construction

The light chain coding sequences were amplified from the cDNA template obtained by RT-PCR from lymphocyte-isolated RNA. The B-cells were obtained from two donors diagnosed of celiac disease during this study and presenting high serum response against gluten proteins, since they had not yet adhered to a gluten-free diet. The first patient showed a 168 UA/mL of IgA response against native gliadin (11 times the baseline of 15 UA/mL) and 550 UA/mL of IgG response against native gliadin (11 times the baseline of 50 UA/mL). The second donor presented 96.5 and 170 UA/mL for IgA and IgG responses, respectively (6.5 and 3.4 times the baselines mentioned above). From the 350 mL of peripheral blood collected from each patient, approximately 1.8 × 10^8^ B-cells (counted with a Neubauer chamber after Trypan Blue staining) were isolated from each sample using a Ficoll gradient. Total RNA was isolated (100 µg) showing high integrity (RIN (RNA integrity number) value of 8.75–9.5 over 10) and used to produce the cDNA of interest by RT-PCR.

The cDNA obtained was used as template for a first round of PCR to amplify the complete light chain coding genes, resulting in amplified fragments of approximately 650 bp. Due to the limited quantity of RNA obtained from the biological samples, only the human kappa chains were amplified in this study, showing a higher expression of the Vk1 set in the electrophoretic analysis (Figure 1B). After gel DNA purification, a second round of PCR was performed to include enhanced restriction sites on the obtained kappa chains for proper cloning [28].

### 3.3. Fab Library Cloning and Panning

For Fab library construction, a two-step cloning process was performed. Firstly, double digested inserts coding for light chains were cloned into linearized pComb3X and transformed into electrocompetent *E. coli* XL-1 Blue, obtaining the light chain sub-library, formed by 1.4 × 10^7^ total transformants. Plasmid DNA was isolated and re-linearized to clone the double digested heavy chain constructs. The final ligation products were precipitated and transformed (elapsed time of 4 ms), resulting in a Fab library with a repertoire size (RZ) of 2.7 × 10^7^ (autoligation factor of 2%), fulfilling the standards considered acceptable for this kind of libraries of RZ > 10^7^ and autoligation factor of 5% [27].

Following helper phage infection, the phage-antibody repertoire was isolated by PEG purification and used for biopanning (successive rounds of selection and amplification) against gliadin-PWG. The repertoire enrichment process was tracked by tittering the output phages after each of the three rounds of selection performed, which corresponds to the infection of the eluted phage-antibodies after antigen exposition. Phage recovery increased 58 times after the third round of panning (Figure S1), indicating the likely selection of phage variants against the target molecule. To further confirm this result, ELISA experiments were developed to evaluate if there was an effective enrichment of the phage population in high-affinity gliadin binders.

### 3.4. Polyclonal Phage-ELISA

The suitability of the panning methodology was checked by ELISA analysis of gliadin-PWG coated plates with the PEG-purified phage repertories (Figure S2). As expected, gliadin was barely detected by the un-selected library (R0), followed by a 7-fold signal increase after the first interaction with the antigen of interest, and maximized absorbance values were obtained with the purified phage populations after the second and third rounds of selection. Very low signals (<0.2 AU) were recorded for the negative control (BSA used as blocking solution) when using the phages obtained after each round of enrichment, indicating that there was no non-specific selection of phage variants against other components of the antigen binding support. Based on these results, a fourth round of selection was considered unnecessary.

### 3.5. Selection of Positive Binders by Monoclonal Phage ELISA

**Figure 2:**
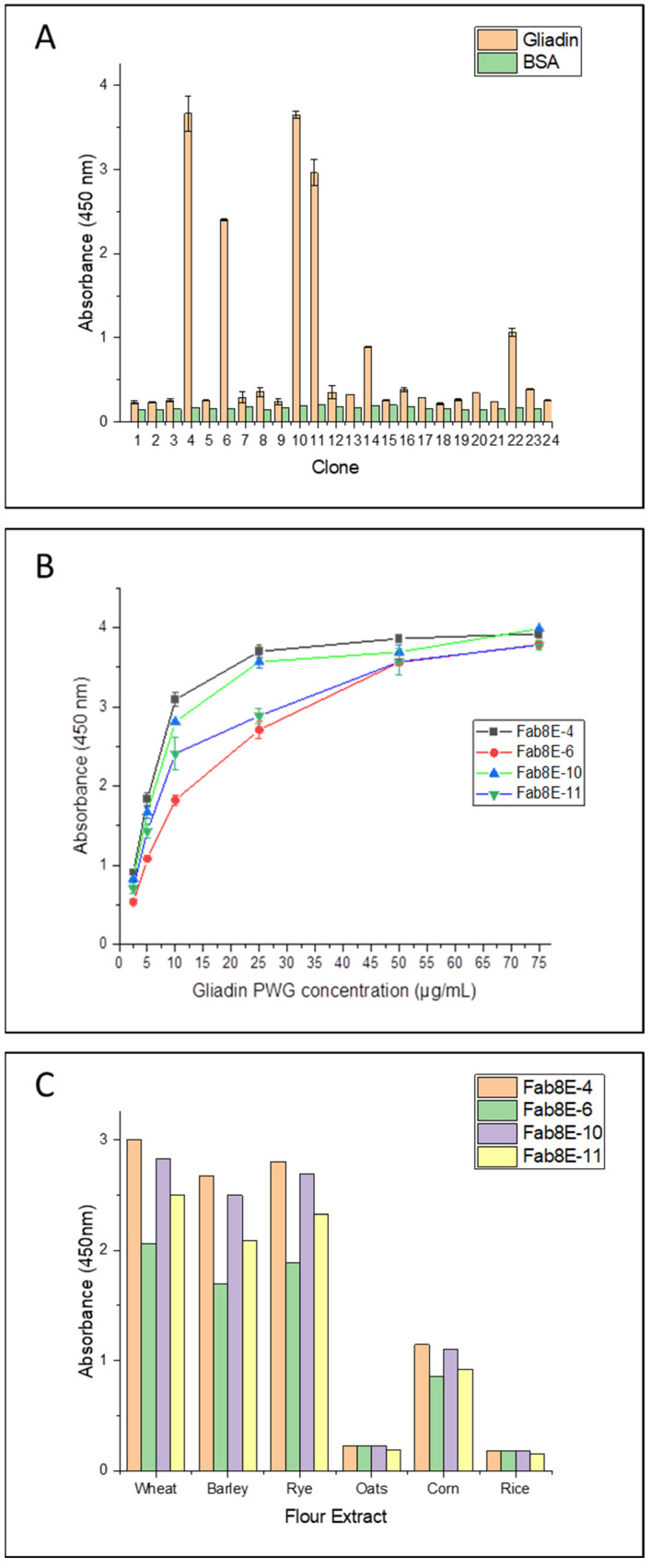
Monoclonal indirect phage-ELISA of Fabs isolated from the panning process. (**A**) Indirect phage-ELISA analysis of individual induced clones isolated from the last round of affinity enrichment. (**B**) Comparison of the dose-response curves by means of the indirect phage-ELISA of the four selected binding clones performed with gliadin-PWG dilutions from 2.5 to 75 µg/mL. Mean values of three independent determinations and standard derivation of each data set are shown. (**C**) Indirect phage-ELISA of positive clones against ethanol protein extracts of different cereal flours (1:100).

A total of 24 individual clones were picked from the tittering plates of the output phage population from round 3. Induced phage supernatants were 1:2 diluted and subjected to monoclonal phage-ELISA in immunoplates coated with gliadin-PWG at 1 µg/well. Predictably, binding and non-binding phage variants were observed, resulting in 4 out of the 24 clones (approximately 17%) selected as high-affinity gliadin-binding clones (absorbance values greater than 2 AU and negative control signals under 0.3 AU) (Figure 2A).

### 3.6. Affinity and Specificity Analysis of the Selected Positive Clones

Monoclonal phage-ELISA experiments were performed to evaluate the affinity and specificity of the four gliadin-binding phage-Fabs. A dose-response curve against increasing concentrations of gliadin-PWG (2.5, 5, 10, 25 and 75 µg/mL) was obtained for each phage variant. Fab8E-4 showed the most promising recognition properties, exhibiting the most sensitive dose-response curve (higher absorbance values at all the gliadin concentrations tested) (Figure 2B).

In addition, a specificity test was conducted with different cereal flours to evaluate the performance of the selected clones in a real food matrix and to discriminate the type of prolamin recognized by the phage-Fabs. As in the case of the gliadin curve analysis, Fab8E-4 showed highest detectability for wheat, barley, and rye prolamins (Figure 2C). Considering these results, among the isolated phage-Fabs, Fab8E-4 presented the best performance for prolamin detection, both in the detection of gliadin dilutions and ethanol extracts from natural gluten sources. Consequently, further characterization experiments were only performed with the phage-Fab8E-4.

### 3.7. Sequencing of Leading Candidates

**Figure 3:**
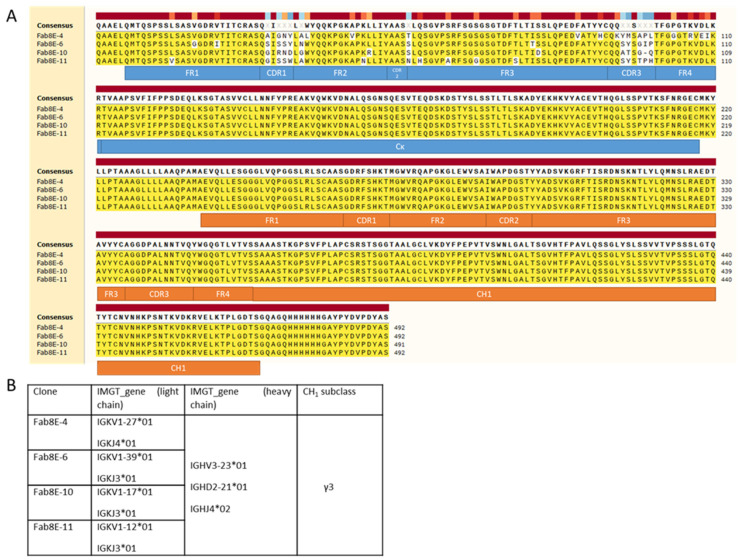
Sequencing results, chain families and features identification of the gliadin-binding selected clones. (**A**) Alignment of the sequences coding for the Fab8E-4, 6, 10 and 11. Main antibody features are shown in blue and orange charts for light and heavy chains, respectively. Alignment was computed using SnapGene^®^ 5.0.8. (**B**) Identification of the re-arranged genes coding the light and heavy chains of the isolated gliadin-binding clones. The VJ and VDJ genes were elucidated using the IMGT^®^/V-QUEST tool, and the IgBLAST tool from NCBI was used for elucidating the CH1 subclass of the heavy chains.

The results of plasmid DNA sequencing of several clones after bacterial transformation showed that the two-step cloning process was successful and that the presence of self-ligation events or other artifacts was not noticeable. Therefore, the constructed library consisted of a Fab repertoire constituted by a collection of light chains expressed in celiac disease patients and the sequence of the dAb-8E transformed into four sets of heavy chains (IgG1, IgG2, IgG3 and IgG4 subclasses). From the DNA sequence analysis of the clones selected as positives after biopanning (Figure 3A), it was inferred that all belonged to different clonotypes derived from the combinatorial arrangement of four different light chains and the same heavy chain, dAb-8E-IgG3 (Figure 3B). The clones selected after three rounds of panning presented light chain diversity (different V and J genes) but only one heavy chain (with the same subclass), this fact could be meaning that the heavy and not the light chains is the principal agent in the molecular evolution process that is selecting the high-affinity clones.

### 3.8. Characterization of Phage Fab8E-4

**Figure 4:**
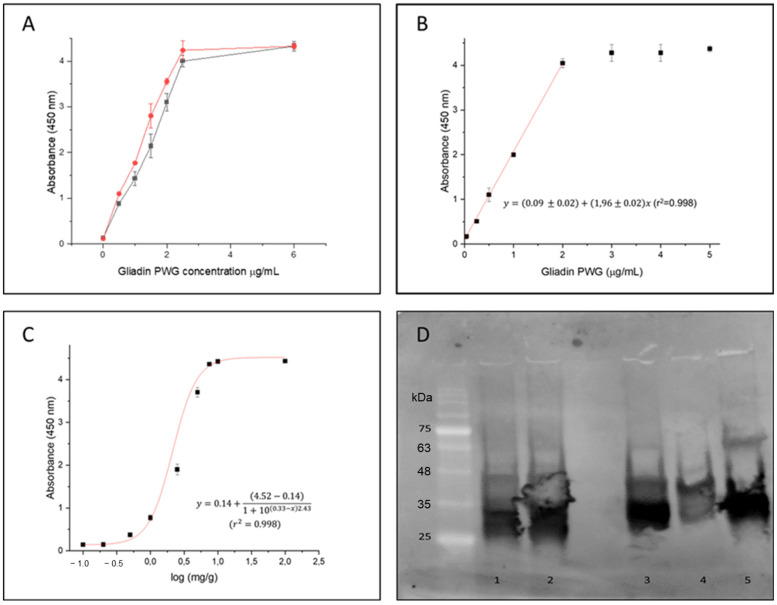
Characterization of phage-Fab8E-4. (**A**) Comparative dose-response curve obtained against gliadin-PWG dilutions (0–6 µg/mL) by means of dAb-8E (grey) and Fab8E-4 (red) antibody fragments attached to VSCM13 helper phage (4 × 10^11^ pfu/well). (**B**) Linear regression obtained against gliadin dilutions (0.01–2 µg/mL) by means of Fab8E-4 in order to determinate the LOD and LOQ. (**C**) Representative dose-response curve from the analysis of experimental rice–based binary mixtures with increasing presence of a proportional mixture of wheat, barley and rye in the range of 0.1 to 100 mg/g. Origin 8.0 software was employed to plot and analyze the experimental data. Mean values of three independent determinations and standard derivation of each data set are shown. (**D**) Western blot analysis of gliadin and flour proteins incubated with phage-Fab8E-4 as primary antibody. Lane 1 (1 mg per well) and Lane 2 (2 mg per well): gliadin (Merck©, CAS #9007–90-3) dissolved in 50% isopropanol-water; Isopropanol extractions of cereal flours: Lane 3: wheat; Lane 4: barley; Lane 5: rye.

In order to compare the sensitivity of the designed phage-ELISA based on the Fab8E-4 with that of the parental phage-dAb8E, both phage-antibody formats were expressed using the same helper phage. For this purpose, it was necessary to produce the dAb-8E attached to the VSCM13 pIII minor capsid protein, instead of the KM13 bacteriophage used before [25]. The comparative dose response curve obtained against increasing concentrations of gliadin-PWG (0.5–6 µg/mL) with both phage-antibody formats is shown in Figure 4A. Following the Eurachem guides for method validation [34], the limit of detection (LOD) was calculated from the interpolation of three times the standard deviation of ten blank determinations in the linear regression in the range 0.01–2 µg/mL (Figure 4B). The LOD for the method using the Fab8E-4 were set at 0.01 µg/mL.

Merging the VL immune chains with the semi-synthetic dAb-8E seemed to slightly improve the LOD for the gliadin reference material solution. However, since immunoassays could be frequently influenced by matrix effects, a closer-to-reality approach was applied. Therefore, a set of binary mixtures was prepared including different spiked levels (0, 0.1, 0.2, 0.5, 1, 2.5, 5, 7.5, 10 and 100 mg/g) of an un-treated wheat/barley/rye proportional mixture (WBRm) in a rice flour matrix [25]. Rice flour was selected as the matrix ingredient of the binary mixture due to the absence of background signal interference when tested in the indirect phage-Fab8E-4 ELISA method and since it is frequently used in gluten-free formulations. Previous studies have adopted a theoretical approach to estimate the amount of gluten as about 10% of the gluten-containing cereal weight [35]. Therefore, the binary mixtures used to assess the suitability of the developed immunoassay to detect low concentrations of gluten in a real food matrix could be interpreted as samples containing approximately 10, 20, 50, 100, 250, 500, 750, 1000 and 10,000 mg of gluten per kilogram (particles per million or ppm of gluten in a food sample). According to these experimental binary mixtures used for in-house validation and to derive the model features, antigen dose-response curve was fitted to a dose response model (Figure 4C). The LOD and LOQ obtained from the analysis of the gluten-spiked mixtures with the developed phage Fab8E-4 assay were 10 and 88 ppm of gluten, respectively. The test seemed to fulfill the legislation requirements, with the ability to distinguish the threshold of 20 ppm used to classify the products as gluten-free or not. The field application functionality of the proposed method was tested with ten food products (five labeled as gluten-free and five as containing gluten) after ethanolic extraction. The indirect phage-ELISA using Fab8E-4 was able to differentiate both types of products. These results were confirmed with a commercial sandwich ELISA based on the R5 monoclonal antibody, the gold standard for gluten analysis (Table S3).

A Western blot analysis was performed to elucidate which prolamin fractions from different gluten-containing cereals were recognized by the developed recombinant antibody (Figure 4D). A commercial preparation of wheat gliadin was used as control because it contains all the different gliadin subunits. The results evidenced that the phage-Fab8E-4 was able to significantly recognize the protein bands between 25 and 48 kDa, that could correspond to the LMW glutenin subunits and alpha-, beta- and gamma-gliadins [35].

### 3.9. Fab8E-4 Sequence Analysis and Structure Prediction

In silico predictions of the Fab8E-4 structure and antibody-antigen interaction were performed to further characterize the antibody fragment obtained.

The library obtained in this work presents half the CDR diversity than a usual immune library, as it applies only to the light chains. Nevertheless, the overall affinity of the library is improved, because many of the phages of a full immune library might not bind properly to the target antigen, while in this library the heavy chains are constituted by dAb-8E gliading-binding chains. Although this situation could lead to miss a “better” new Fab that could be found, or not, in a classical immune library, the selection of a minor expressed class, like IgG3, shows that the artificial rearrangement could generate functional structures that are very underrepresented in nature. This improvement is paired with an increase of Fab8E-4 affinity and avidity to gliadin, which derives from the new structure.

**Figure 5:**
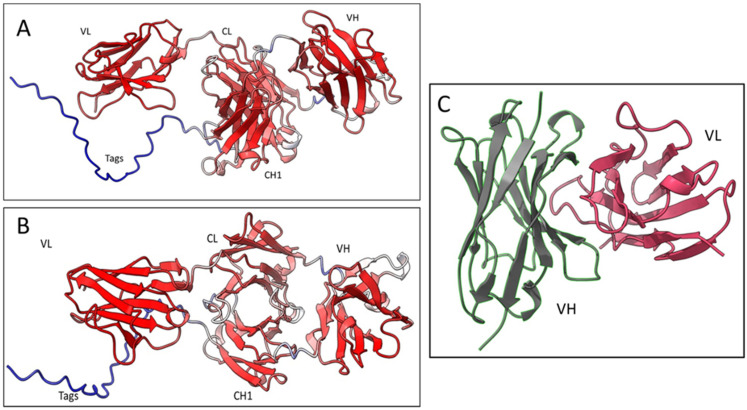
Computational models of the Fab8E-4. (**A**) Predictive model of Fab8E-4 generated with Alpha-Fold2. The pLDDT scores are shown with the following scale of color red > white > blue. (**B**) Same Alpha-Fold2 predicted model rotated for better visualization of the different domains. (**C**) Fv predictive model generated by Antibody-builder (sAbpred server).

Based on the sequencing results of the Fab8E-4, the whole molecule 3D-structure prediction was generated using Alpha-fold2, an artificial intelligence program that performs predictions of protein structure with high accuracy [36]. Five different models were generated, and each model was recycled three times. The models were ranked by pLDDT score (a per-residue confidence estimation on a scale of 0–100). The fifth model (Figure 5A and 5B) was selected as the best, with an overall pLDDT score of 82,3 which was considered a good backbone prediction [37]. Besides, taking into account that the worst resolved area was the histidine tag attached to the CH1 domain which lowered the overall score, the Fab8E-4 domains were fairly well resolved with pLDDT scores >85.

In addition to the whole molecule model, a second model of the Fab was generated focused on the Fv (fragment variable of the antibody, composed of the VL and VH), the main antigen-antibody interaction surface. This model was computed using the AbodyBuilder tool included in the sAbPred server (Figure 5C). This bioinformatic tool encompasses up to seven different algorithms for the determination of the most likely antibody structure, such as identification of the best templates for VH and VL domains, prediction of the VH-VL orientation or modeling of CDR-loops and side chains. The background structures that were chosen as framework templates were (RCSB PDB identifiers) 6ghg (VH) and 6co3 (VL) as they showed the highest sequence identity for each chain (86.2% sequence identity for VH and 93.4% for VL). The VH-VL orientation was predicted using the angles inherited from the background structure 2uzi.

Once the 3D model of the Fab8E-4 was obtained, epitope and paratope prediction was assessed to investigate the antigen-antibody interaction surface. To identify the main amino acids involved in the binding region, knowledge of the 3D structure of both, the antigen and the Fv of the Fab, is required. However, crystal structures of the different gluten proteins were not available in databases such as Uniprot or RSCB PDB (which only include structures from some linear peptides presented by HLA that were not useful for the present work). Despite the lack of experimental data on crystal gluten structures, the structures of some gliadins and other gluten-related proteins have been recently solved with AlphaFold, and uploaded in Uniprot database. Therefore, in this study epitope and paratope predictions were performed with Epipred and Antibody-ipatch algorithms, respectively, using as protein templates the 3D antibody structure generated by AbodyBuilder and the antigen 3D-structure of wheat α/β-gliadin and barley γ-hordein solved by AlphaFold (P18573 and P17990 Uniprot entries). These entries were chosen as they showed the highest quality of information recorded in the database. Until the structure of gliadins are not completely solved by X-ray crystallography or cryomicroscopy, this approach was chosen as the most adequate approximation for epitope and paratope identification of Fab8E-4.

**Figure 6:**
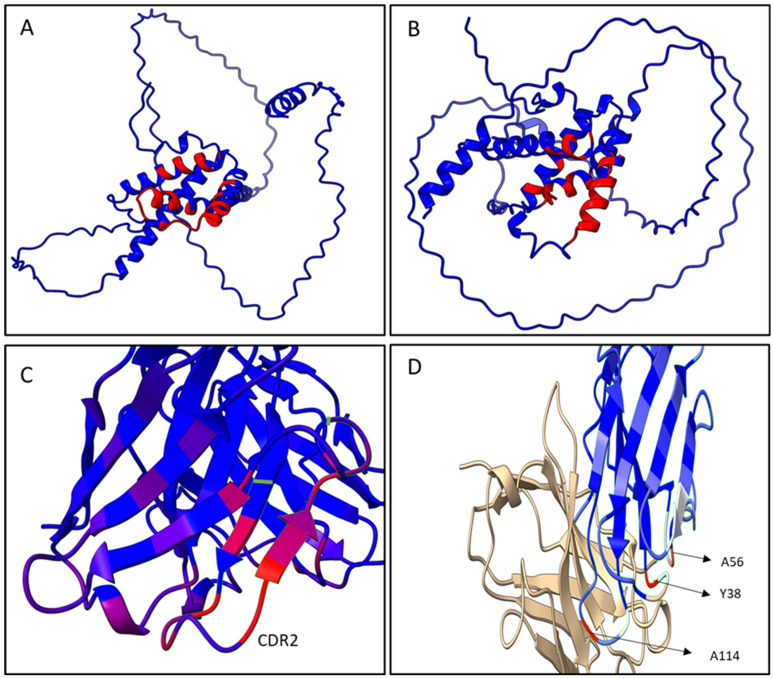
Epitope prediction (**A**,**B**) in α/β-gliadin (**A**) and γ-hordein (**B**) facing Fab8E-4. The residues colored in red are more likely to be part of the conformational epitope. Notice that the images have been rotated to show the conformational epitope. Models generated with EpiPred (SAbPred server). Paratope prediction in Fab8E-4 (**C**,**D**). The residues are colored according to the likelihood of being part of the paratope following the order: red >white >blue. Notice that the images have been rotated to show the zones with a higher likelihood of being part of the paratope. The model was generated with antibody i-patch (SAbPred server). (**C**) In red the residues of the heavy chain identified to be more likely to interact with the antigen. CDR2 and neighboring sequences in a green silhouette. (**D**) In red the residues of the light chain identified to be more likely to interact with the antigen.

The epitope predictive models depicted in Figure 6A,B show that the conformational epitopes were mainly found in the α-helix motifs on the C-terminal region of both antigens, the α/β-gliadin and the γ-hordein. Within the gliadins studied, the main interaction zone of the dAb-8E and Fab8E-4 is located downstream one of the most pathogenic reported zones for celiac disease [38]. Besides, it presented more dispersed and shorter repeats of glutamines than those found in the N-terminal half. This fact could be explained because the heavy chain was originally derived from a semi-synthetic library instead of an immune one, which presumably would have resulted in an antibody binding the pathogenic N-terminal segment. In this sense, Fab8E-4 and their parental antibody fragment dAb-8E constitute a new alternative for gluten determination, providing likely contacts to the C-terminal region of the antigens, which could complement the already available antibodies that usually bound to the N-terminal region. This opens the possibility of further developments of sandwich and competitive ELISAs with antibodies binding different epitopes.

As shown in the paratope models obtained in Figure 6C,D, regardless the prolamin used in the analysis of the paratope models, the main antigen-antibody interaction remained in the heavy chain of Fab8E-4, which was derived from dAb-8E. The residues most likely to be in contact with the antigen were in the CDR-2 and neighboring heavy chain sequences (Figure 6C). However, reformatting of the dAb-8E added new strong contacts with the antigen through several light chain residues (A56, Y38 and A114), which presumably could have improved the characteristics of the detection method. In addition, the disulfide bond between the constant and the variable domains fixed the structure, reducing the conformational freedom of the protein [27].

## 4. Conclusions

This work presents a novel approach to generate a suitable library for selection of antibody fragments for prolamin detection. This method goes beyond the direct transformation of an antibody format to another (dAb into Fab), implying a direct evolution process in order to improve the features of the previously obtained dAb-8E. A library composed of light chains and heavy chains from different origin was built. The designed SOE-PCR reactions allowed a quick conversion of the dAb sequence into a Fab heavy chain and, once cloned into the subset of light chains obtained from celiac donors, the library was obtained. The high diversity of the constructed library resulted in the successful isolation of positive clones. After characterization of different phage variants, the Fab8E-4 was selected and tested in an immunoassay for the specific and sensitive detection of gluten. Besides, a deeper study of the generated antibody was developed using in silico tools. The proposed ELISA method demonstrated that transformation of the dAb-8E into a Fab structure resulted in an improvement of the characteristics of the developed methodologies for gluten detection in foodstuff. Therefore, the obtained Fab8E-4 presented enhanced properties regarding the parental dAb, indicating that the proposed methodology was a useful tool to improve the performance of existing antibodies.

The phage-Fab8E-4 obtained and characterized in this work has demonstrated ability to detect gliadin in solution, an also in complex mixtures. Thus, it can be directly applied for gluten detection in foodstuff. Moreover, thanks to the in silico model, that showed contacts with the C-terminal gliadin portion, it could be used as a complementary or alternative reagent to the other antibodies in the market for gluten detection, that bind to the N-terminal portion of the antigen.

The development of gluten detection assays has been historically focused on hybridoma generated monoclonal antibodies (R401, R5, G12). Seldom recombinant antibodies have been used for solving this problem, and mainly they were applied for studying clinical features of gluten-related diseases rather than gluten detection in foods. The present work is a novel rational approach that goes beyond the construction of an immune library from celiac patients. The construction of a novel Fab library is provided, beginning with the scaffold of a pre-existing antibody selected against likely pathogenic gluten peptides, transformed into a Fab heavy chain, and merged with a light chain subset derived from celiac patients. This is the first time a directed evolved engineered antibody fragment has been applied for gluten detection in foodstuffs.

## Data Availability

Data is contained within the article.

## Supplementary Materials

The following supporting information can be downloaded at: https://www.mdpi.com/article/10.3390/foods12010149/s1, Figure S1: Transformants recovery after panning rounds; Figure S2: Indirect phage-ELISA analysis of the phage-Fab library rescued after each round of panning; Table S1: Primers used for heavy chain amplification; Table S2: Primers used for light chain amplification; Table S3: Sequencing primers; Table S4: ELISA results obtained for detection of gluten with the phage Fab8E-4 and R5 monoclonal antibodies from ethanolic extracts of 10 commercial food products.
