# Supplementary material for: Construction of a Fab Library Merging Chains from Semisynthetic and Immune Origin, Suitable for Developing New Tools for Gluten Immunodetection in Food
Source: Foods. 2022 Dec 28;12(1):149. doi: 10.3390/foods12010149 (PMC9818130; doi:10.3390/foods12010149)
Supplement: Supplementary file 1 [file foods-12-00149-s001.zip › foods-2045507-supplementary.pdf]

Table S1. Primers used for heavy chain amplification

Name	Sequence
**First PCR round**	
VH135 fw	AGG TGC AGC TGC TCG AGT CTG G
ovlp-conga rev	AAG ACC GAT GGG CCC TTG GTG GAT GCG GCC GCG CTC GAT ACG G
ovlp-cong4 rev	ACG GAT GGG CCC TTG GTG GAA GCT GCG GCC GCG CTC GAT ACG G
**Second PCR round**	
VH135ext fw	(GTC)7 G TAG GTG CAG CTG CTC GAG TC
CG1Zext rev	(GA)10 G GCA TGT ACT AGT TTT GTC A C
CG2a ext rev	(GA)10 CTC GAC ACT AGT TTT GCG
CG3a ext rev	(GA)10 TGT GTG ACT AGT GTC ACC
CG4a ext rev	(GA)10 GCA TGA ACT AGT TGG GGG

Table S2. Primers used for light chain amplification

Name	Sequence
**First PCR round**	
Vκ1 fw	GAG CCG CAC GAG CCC GAG CTC CAG ATG ACC CAG TCT CC
Vκ2/4 fw	GAG CCG CAC GAG CCC GAG CTC GTG ATG ACY CAG TCT CC
Vκ3 fw	GAG CCG CAC GAG CCC GAG CTC GTG WTG ACR CAG TCT CC
Vκ5 fw	GAG CCG CAC GAG CCC GAG CTC ACA CTC ACG CAG TCT CC
Cκ1d rev	GCG CCG TCT AGA ATT AAC ACT CTC CCC TGT TGA AGC TCT TTG TGA CGG GCG AAC TCA G
**Second PCR round**	
Vκ ext fw	GCG CCG TCT AGA ATT AAC ACT CTC CCC TGT TGA AGC TCT TTG TGA CGG GCG AAC TCA G
Cκ ext rev	GAG GAG GAG GAG GAG GAG GCG CCG TCT AGA ATT AAC ACT CTC

Table S3. Sequencing primers

Name	Sequence
ompAseq	aagacagctatcgcgattgcag
g-back	gcccccttattagcgtttgccatc

Degenerated bases: W=A,T; R=A, G.

Table S4. ELISA results obtained for detection of gluten with the phage Fab8E-4 and R5 monoclonal antibodies from ethanolic extracts of 10 commercial food products.

Nº	Sample	Gluten Label	Fab8E-4	R5
1	Macarrons (Bakery)	+	+	+
2	Apple cake	+	+	+
3	Free-sugar biscuits	+	+	+
4	Caramelized biscuits	+	+	+
5	Cake	+	+	+
6	Sausages	-	-	-
7	Vegan sausages	-	-	-
8	Surimi	-	-	-
9	Nuts mixture	-	-	-
10	Sunflower seeds	-	-	-

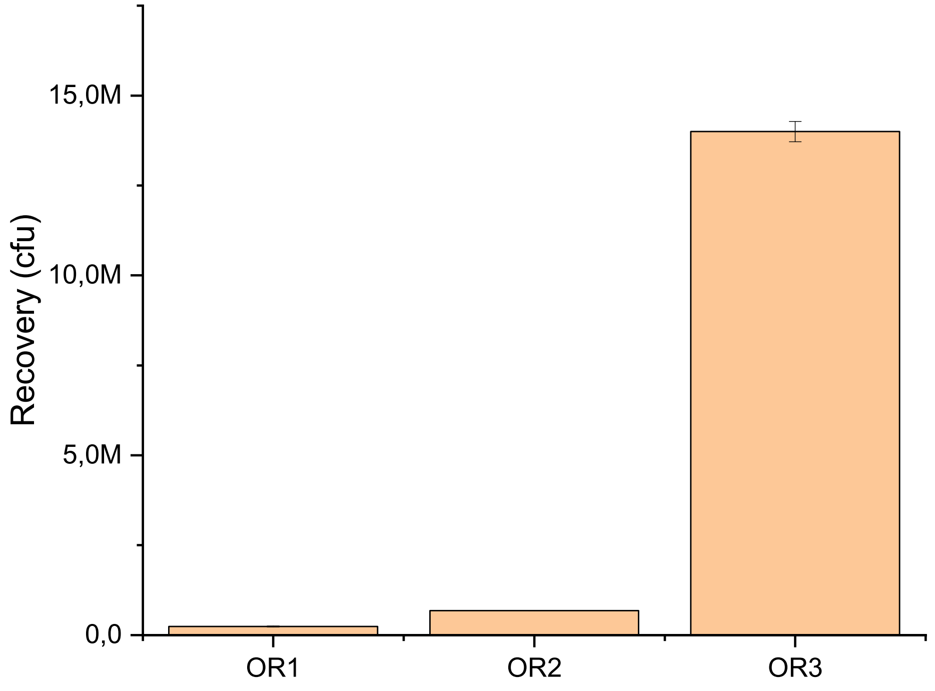

Figure S1. Recovery of transformants after each round of panning. An inference was calculated from the colonies grown after plating in SB-carbenicilin (100 µg/mL) infected *E. coli* XL-1 with the output phages from each round of selection: Output of Round 1 (OR1); Output of Round 2 (OR2); Output of Round 3 (OR3). Mean values of two independent determinations and standard derivation of each data set are shown.

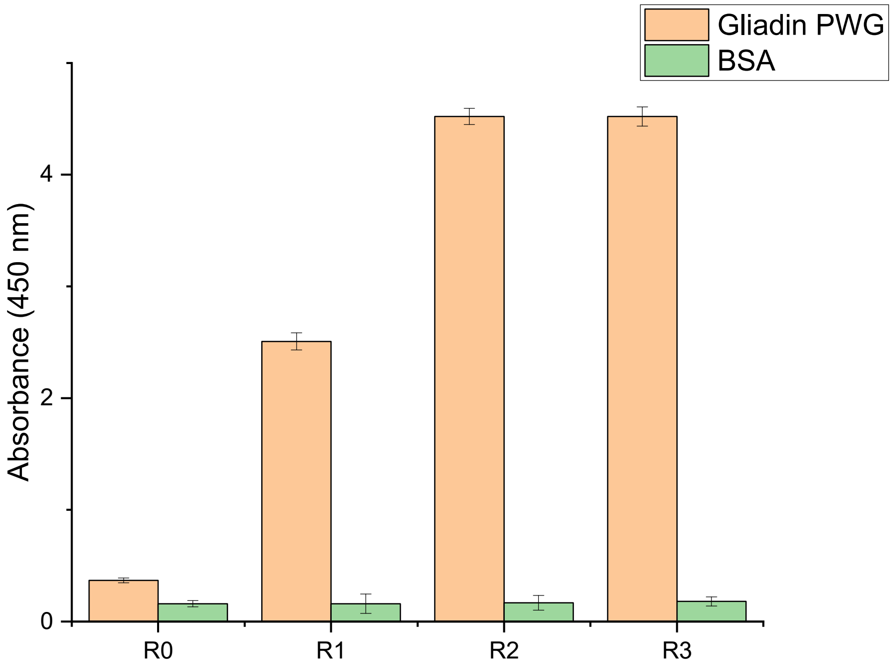

Figure S2. Indirect phage-ELISA analysis of the phage-Fab library rescued after each round of panning. Results of absorbance value (450 nm) obtained against gliadin-PWG (objective antigen) and BSA (blocking solution). Mean values of three independent determinations and standard derivation of each data set are shown.
